# An implementation intervention to increase the routine provision of antenatal care addressing gestational weight gain: study protocol for a stepped-wedge cluster trial

**DOI:** 10.1186/s43058-021-00220-y

**Published:** 2021-10-19

**Authors:** Melanie Kingsland, Jenna Hollis, Eva Farragher, Luke Wolfenden, Karen Campbell, Craig Pennell, Penny Reeves, Belinda Tully, Justine Daly, John Attia, Christopher Oldmeadow, Mandy Hunter, Henry Murray, Francesco Paolucci, Maralyn Foureur, Chris Rissel, Karen Gillham, John Wiggers

**Affiliations:** 1 Hunter New England Population Health, Hunter New England Local Health District, Wallsend, New South Wales Australia; 2 School of Medicine and Public Health, College of Health, Medicine and Wellbeing, The University of Newcastle, Callaghan, New South Wales Australia; 3 Hunter Medical Research Institute, New Lambton Heights, New South Wales Australia; 4 Priority Research Centre in Health Behaviour, The University of Newcastle, Callaghan, New South Wales Australia; 5 Institute for Physical Activity and Nutrition, School of Exercise and Nutrition Sciences, Faculty of Health, Deakin University, Melbourne, Victoria Australia; 6 Department of Maternal Fetal Medicine, Maternity and Gynaecology, John Hunter Hospital, New Lambton Heights, New South Wales Australia; 7 Nursing and Midwifery Services, Hunter New England Local Health District, New Lambton Heights, New South Wales Australia; 8 Faculty of Business and Law, The University of Newcastle, Newcastle, New South Wales Australia; 9 The School of Economics and Management, University of Bologna, Bologna, Italy; 10 School of Nursing and Midwifery, College of Health, Medicine and Wellbeing, The University of Newcastle, Newcastle, New South Wales Australia; 11 Centre for Midwifery, Child and Family Health, Faculty of Health, University of Technology Sydney, Ultimo, New South Wales Australia; 12 Hunter New England Health Nursing and Midwifery Research Centre, Newcastle, New South Wales Australia; 13 The Australian Prevention Partnership Centre, Sax Institute, Sydney, New South Wales Australia; 14 Flinders University, Darwin, Northern Territory Australia; 15 Early Prevention of Obesity in Childhood Centre for Research Excellence, Sydney, New South Wales Australia

**Keywords:** Pregnancy, Weight, Physical activity, Nutrition, Antenatal, Implementation, Effectiveness, Stepped-wedge trial, Protocol

## Abstract

**Background:**

Weight gain during pregnancy that is outside of recommended levels is associated with a range of adverse outcomes for the mother and child, including gestational diabetes, pre-eclampsia, preterm birth, and obesity. Internationally, 60–80% of pregnant women report gaining weight outside of recommended levels. While guideline recommendations and RCT evidence support the provision of antenatal care that supports healthy gestational weight gain, less than 10% of health professionals routinely weigh pregnant women; discuss weight gain, diet, and physical activity; and provide a referral for additional support. This study aims to determine the effectiveness of an implementation intervention in increasing the provision of recommended gestational weight gain care by maternity services.

**Methods:**

A stepped-wedge controlled trial, with a staggered implementation intervention, will be conducted across maternity services in three health sectors in New South Wales, Australia. The implementation intervention will consist of evidence-based, locally-tailored strategies including guidelines and procedures, reminders and prompts, leadership support, champions, training, and monitoring and feedback. Primary outcome measures will be the proportion of women who report receiving (i) assessment of gestational weight gain; (ii) advice on gestational weight gain, dietary intake, and physical activity; and (iii) offer of referral to a telephone coaching service or local dietetics service. Measurement of outcomes will occur via telephone interviews with a random sample of women who attend antenatal appointments each week. Economic analyses will be undertaken to assess the cost, cost-consequence, cost-effectiveness, and budget impact of the implementation intervention. Receipt of all care elements, acceptance of referral, weight gain during pregnancy, diet quality, and physical activity will be measured as secondary outcomes. Process measures including acceptability, adoption, fidelity, and reach will be reported.

**Discussion:**

This will be the first controlled trial to evaluate the effectiveness of a implementation intervention in improving antenatal care that addresses gestational weight gain. The findings will inform decision-making by maternity services and policy agencies and, if the intervention is demonstrated to be effective, could be applied at scale to benefit the health of women and children across Australia and internationally.

**Trial registration:**

Australian and New Zealand Clinical Trials Registry, ACTRN12621000054819. Registered on 22 January 2021

Contributions to the literature

- This is the protocol for the first controlled trial of an implementation intervention to support antenatal clinicians improve care for women for gestational weight gain.
- The implementation intervention will use innovative best-worst scaling methods to assess the priority barriers of clinicians and develop tailored implementation strategies specific to the needs of each sector.
- Data from approximately 5600 women will measure care-level outcomes to determine if the implementation intervention was successful. This will be supplemented by an assessment of cost-effectiveness, cost-consequence, and budget impact; diet, physical activity, and weight outcomes for women; and process measures including acceptability and adoption.
- These comprehensive outcomes will provide a robust assessment of the implementation intervention and its ability to change clinician’s practices to benefit the health of women and their babies. This will provide maternity services and policymakers with evidence to support such an intervention being supported for further scale-up.

## Background

Gestational weight gain (GWG) below or above-recommended levels is a leading risk factor for poor pregnancy outcomes, with the potential for harm to both mother and child [1]. For the mother, these include a higher risk of gestational diabetes [2], caesarean birth [1], greater postpartum weight retention, and greater risk of obesity long term [3–5]. For the baby, excess gestational weight gain is associated with a higher risk of macrosomia and neonatal morbidity [6].

To quantify an ideal gestational weight gain range, in 2009, the United States Institute of Medicine (IOM) developed total and trimester-specific incremental weight gain recommendations for optimal healthy weight gain during pregnancy based on a woman’s pre-pregnancy body max index (BMI) [7]. Guidelines in Australia [8] and other high-income countries [7, 9] recommend that antenatal care providers support women to gain weight within these recommendations. Internationally, most (60–80%) women gain weight outside of these recommended target ranges [1]. In Australia, 64% of pregnant women report gaining weight outside of recommendations [10], including 85% of pregnant Aboriginal Australian women [11]. A systematic review and meta-analyses of studies including over 1.3 million pregnancies found that of the women who gained weight outside of the IOM recommended ranges, 9.5–29% gained below and 37–73% above the recommended ranges [1, 12, 13].

Cochrane systematic review evidence shows that educational and behavioural interventions supported by weight monitoring are effective in reducing the risk of unhealthy gestational weight gain by 20% (average RR 0.80, 95% CI 0.73 to 0.87) [14]. Systematic review evidence also suggests that such interventions are most effective when delivered as part of routine antenatal care [15], and it has been reported that women prefer such care to be provided in a way that is person-centred and non-judgemental [16].

Consistent with this evidence, antenatal care guidelines in countries including Australia [8], Canada [17], New Zealand [18], and Ireland [19] recommend that antenatal care providers routinely (i) assess weight and gestational weight gain of all women at all antenatal visits; (ii) provide brief behavioural support to all women addressing weight gain, healthy eating, and physical activity; and (iii) offer referral to specialist services for support for weight gain, healthy eating, and physical activity, particularly for those women who are gaining weight outside their weight gain target.

Despite such guidelines, the provision of antenatal care supporting healthy gestational weight gain has been reported to be less than optimal [20]. For instance, in Canada, it has been reported that only 16% of health professionals (*N* = 508) routinely discuss a woman’s weight gain during antenatal visits, and just 28% and 46% discuss healthy eating and physical activity, respectively [21]. Additionally, a 2009–2010 secondary analysis of data from antenatal visits in the United States (U.S.) found that the majority of visits (59%; *N* = 120) included only one of five evidence-based elements of care related to weight, and no visit included all five [22]. Forty-nine per cent of women had their weight gain assessed, 85% received weight gain advice, 3% received assistance to achieve the recommended weight gain, and 11% had follow-up care arranged [22]. Similarly, of the 1454 women in a longitudinal cohort study in a hospital in North Carolina, USA, just over half (52%) reported receiving advice about gestational weight gain from their health care providers [23].

A variety of barriers impede the provision of recommended gestational weight gain care [20, 21], including forgetting to undertake assessments and care, inadequate knowledge of guidelines, concerns about women’s sensitivity to discussing weight, and lack of time, resources and referral sources, skill, and understanding of the need to provide such care [20, 24]. Systematic reviews suggest that a variety of strategies are effective in addressing such barriers to improving the provision of guideline-based care generally [25] and in maternity services specifically [26]. Such strategies include clinical leadership [27], systems and policies that support/prompt care delivery [28], clinician training [29–31], and audit and feedback of care delivery [26, 31, 32]. Across a variety of clinical settings, such strategies can result in a 5–20% improvement in the delivery of desired clinical practices [25]. Tailoring of these strategies to local barriers and local context through the use of theoretical implementation science frameworks and associated tools [33, 34] has been found to further aid the development of successful practice change interventions [35, 36].

There have been a small number of studies undertaken in single maternity services that have assessed the effectiveness of practice change strategies to improve the provision of weight-related care in pregnancy [37–41]. These controlled and uncontrolled trials used multiple evidence-based strategies including education and training, clinical supervision, local care guidelines, provision of clinical equipment and resources, and medical record prompts to support health professionals to support practice change [37–39, 41], with most testing a combination of clinician training, clinical equipment, and patient resources. However, only one applied a theoretical framework and conducted systematic barrier analysis and mapping as a basis for strategy development [41].

In addition, no studies have tested the effect of practice change strategies to enhance all elements of antenatal guideline care for gestational weight gain, that is, assessment of weight, provision of advice, and referral to support services [41]. Most existing studies have examined the effect of strategies on routine weight assessment only, provision of advice for gestational weight gain only, or both assessment and advice. For example, an Australian cohort study (*N* = 13,000 pregnancies) found that recording of weight improved through the provision of weighing scales and staff training (18.9%) and medical record prompts (61.8%) (*p* < 0.01) [41]. A U.S. cohort study of 733 clinicians that introduced medical record prompts also demonstrated an increase in recording of weight (*p* < 0.001) and GWG counselling (*p* < 0.001) [42].

Similarly, despite intervention cost-effectiveness and budget impact being a significant factor in health care decision-making [43], no economic evaluations of such implementation interventions have been reported [44]. Given these evidence gaps, a need and opportunity exist for rigorous trials to examine the effectiveness, efficiency, and affordability of implementation interventions designed to increase the provision of recommended antenatal care addressing gestational weight gain.

## Methods

See ‘Additional File 1’ and ‘Additional File 2’ for the CONSORT (stepped-wedge trial) and STARi checklists.

### Aim

The aim of this study is to determine the effectiveness of an implementation intervention in increasing the provision of recommended gestational weight gain care in antenatal appointments.

### Study design and setting

**Fig. 1:**
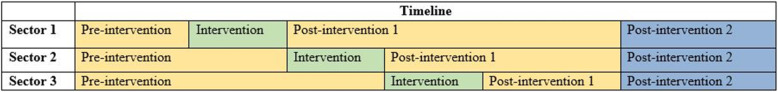
Data collection and intervention timeline for the stepped-wedge trial design

A stepped-wedge controlled trial will be conducted in maternity services in three health sectors within the Hunter New England Local Health District, New South Wales, Australia. As shown in Fig. 1, measurement of the outcomes will occur continuously via telephone surveys with weekly random samples of pregnant women who have attended the maternity services in the last 2–3 weeks, from 6 months prior to the delivery of the intervention in the first sector to 12 months after intervention completion in the last sector. Delivery of the 4-month implementation intervention in the three sectors will occur sequentially. The sequential delivery will provide pre- and post-intervention outcome data periods of variable lengths for each sector, with a minimum of 12 months post-intervention data to enable the assessment of the sustainability of the intervention effect. Intervention effect will be determined by comparing the prevalence of recommended care between the pre-intervention and post-intervention periods for the three sectors combined.

The stepped-wedge study design provides pragmatic and scientific advantages relevant to the conduct of complex implementation interventions in health services [32, 45]. First, such a design provides the same level of evidence as a standard parallel cluster controlled trial [46]. Second, the design addresses the practical difficulty of recruiting enough equivalent maternity services that would be required for a parallel cluster RCT and increases study efficiency by using each group as its own control [32, 45]. Third, the design provides an opportunity for all participating services to receive the intervention, providing motivation for clinician engagement. Finally, such a design demonstrates the feasibility of the intervention in an operational environment, a key determinant of the intervention being translated into routine practice elsewhere should it prove successful [45].

In Australia, public maternity services are a key setting for the provision of antenatal care to address gestational weight gain as they provide care for 55% of all birthing women, including care for a diverse range of population groups, including those with a high prevalence of risk and those who are disadvantaged [47, 48]. The maternity services involved in this trial provide care to approximately 7000 women annually across urban and rural areas, accounting for almost 70% of women birthing in public hospitals within the health district [46].

### Participant blinding

Study personnel involved in collecting the outcome data will be blind to the order of the delivery of the implementation intervention across the sectors. Participants providing outcome data will not be informed of the experimental nature of intervention implementation across services and therefore will be blind to the stage of intervention occurring in the service they attended. Given the practice change nature of the intervention, clinicians in antenatal services will be aware when their service is in the intervention period.

### Participant eligibility and recruitment

#### Maternity services and clinicians

All health professionals who provide antenatal care within the participating maternity services will be targets to receive the implementation intervention, including registered midwives (clinical midwife educators, clinical midwife specialists, clinical midwife consultants, community liaison midwives), medical practitioners (staff specialists in obstetrics, fellows, registrars, resident medical officers, general practice obstetricians), Aboriginal Health Practitioners, Aboriginal Health Workers, and students. All such clinicians who work in participating maternity services at any time during the 4 months when the implementation intervention is delivered in their sector will be invited to participate in a post-intervention survey.

#### Pregnant women

All pregnant women who attend participating services from the start of the implementation intervention in their sector will receive the recommended model of gestational weight gain care. During the 30-month data collection period, women who have had an antenatal appointment at any of three time points: (i) the first public antenatal clinic appointment, (ii) 27–28 weeks gestation, or (iii) 35–36 weeks gestation, will be eligible to participate in the data collection surveys. Eligibility for participation in such surveys will require women to be aged 18 years or older, be pregnant at more than 12 weeks gestation and less than 37 weeks gestation, have a sufficient level of English language proficiency to complete the survey unaided, and be mentally and physically capable of completing the survey. Women will be ineligible to participate in the data collection surveys if they are determined by a maternity service clinician to be inappropriate to contact for the survey (e.g. due to medical or social issues), are seeing a private provider as the primary provider of their antenatal care, have given birth or had a negative pregnancy outcome, had already been selected to participate in the survey for that time point in the past 4 weeks, and/or had previously declined participation in the survey. Characteristics of women deemed ineligible will be recorded and reported.

Each week, a sample of eligible women who have had an antenatal appointment in the past week (first appointment, 27–28 weeks gestation, or 35–36 weeks gestation) will be randomly selected via a computerised random number generator by members of the research team, none of whom will be involved in delivering antenatal care. Selected women will be mailed a participant information statement explaining the purpose of the survey one week prior to receiving a phone call inviting them to participate in the survey. Study posters will be displayed in antenatal clinics and flyers distributed in antenatal information packs provided at the time of the appointment. Based on cultural advice, women identified via the medical record data as being of Aboriginal or Torres Strait Islander origin and/or who are enrolled in an Aboriginal Maternal and Infant Health Service (AMIHS) will be first contacted by text message and invited to participate. If potential participants do not respond, they will be followed up with a telephone call 4 days later. Sampled women will have the opportunity to decline participation at any point, including opting out during the antenatal appointment or when they receive study information during their appointment, when they receive the study information letter in the mail, at the time of the phone call or text message, or partway through survey completion. On the morning of the day that contact is to be made via phone call or text message, medical record data will be checked, and any potential participants who are identified as having given birth or having had a negative pregnancy outcome will be deemed ineligible and not contacted.

### Intervention

#### Best practice care pathway for addressing gestational weight gain

**Fig. 2:**
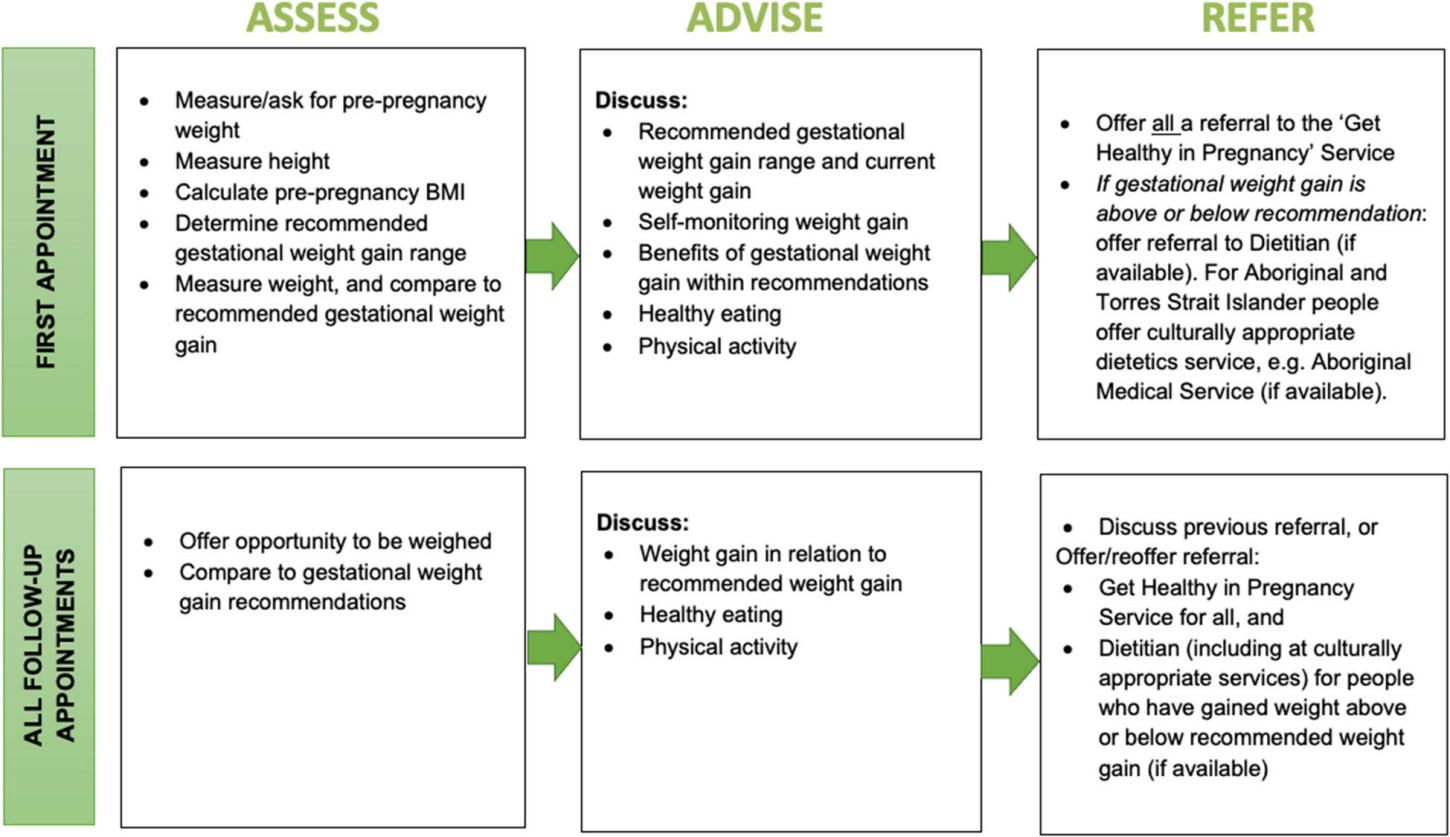
Gestational weight gain care pathway

A best practice care pathway for addressing gestational weight gain in pregnancy will be implemented in maternity services across the three participating sectors. The gestational weight gain care pathway is consistent with international [9] and Australian national [8] and state [49] antenatal clinical practice guidelines and is based on models of assessment and brief intervention that have been shown to support weight gain within recommended ranges [14, 15]. As shown in Fig. 2, the gestational weight gain care pathway will consist of three key care elements—assessment, advice, and referral—delivered during antenatal appointments throughout pregnancy via face-to-face, telephone, and/or video conference consultations.

##### Assessment of gestational weight gain

At the first antenatal appointment, a woman’s pre-pregnancy BMI will be calculated based on reported pre-pregnancy weight (or weight measured at an early pregnancy appointment), and height measured at the appointment. Using their pre-pregnancy BMI, recommended weight gain targets for pregnancy will be determined using IOM recommendations [7]. At each subsequent face-to-face antenatal appointment, women will be offered the opportunity to be weighed. At appointments that are not face-to-face, women will be asked to report their current weight if they have access to weighing scales. Their weight at each appointment will be compared to their recommended weight gain target for their current gestation.

##### Verbal advice on gestational weight gain, diet, and physical activity

At all antenatal appointments, there will be a discussion with women about their gestational weight gain, diet, and physical activity. Verbal and written [50] advice on gestational weight gain will be provided based on their current gestational weight gain relative to their gestational weight gain target, including discussions on the benefit of gestational weight gain within recommendations. All women will be encouraged to self-monitor their weight gain during pregnancy using the NSW Get Healthy in Pregnancy online weight gain calculator [51]. Advice will be provided on healthy eating consistent with the Australian Dietary Guidelines [47], with a pregnancy-specific pamphlet provided to women [52, 53]. Physical activity recommendations will be provided consistent with the Australian government guidelines for adults during pregnancy [48], with a pregnancy-specific pamphlet provided to women [54].

##### Referral to services for additional support for gestational weight gain, diet, and physical activity

All women will be offered a referral to the Get Healthy in Pregnancy (GHiP) telephone coaching service, a free, state-wide, government-funded service [55, 56]. GHiP participants can choose to focus on weight gain and/or physical activity and/or dietary intake and receive up to 10 tailored calls by qualified health coaches throughout their pregnancy. The coaching is based on goal setting, motivation, and overcoming barriers [55]. For women who are gaining weight above or below their recommended weight gain target, an offer of a referral to a dietetics service will also be provided, where such local services are available. For women who identify as Aboriginal and/or Torres Strait Islander, referral options will include dietetics services within Aboriginal Community Controlled Health Services, where available.

#### Implementation intervention

The following evidence-based organisational and individual clinician focused strategies will be used to support clinicians deliver the gestational weight gain care pathway as part of routine antenatal care. The specific content of each strategy and the prioritisation of resources allocated to the strategies will be tailored to the needs and context of each individual sector.

1. *Leadership* [27]: Existing clinical networks and maternity service clinical leaders will be engaged to facilitate ongoing authorisation and endorsement of the initiative. This will include the presence of clinical leaders at training and in communicating performance feedback to staff members. Clinical leadership groups in each sector will provide clinical guidance across all components of the intervention. Aboriginal staff and partners will provide oversight of the cultural appropriateness of the intervention.
2. *Local guidelines and procedures* [28]: Will be developed to outline the care pathway elements, including local service referral options and procedures specific to each maternity service.
3. *Prompts and reminders* [57]: Physical point-of-care prompts including stickers in the antenatal care record, and a clinic room flip chart, will be provided to prompt recommended care delivery.
4. *Service champion* [58, 59]: A dedicated Clinical Midwife Educator (CME) will be employed in each of the three health sectors for the 4-month intervention phase to facilitate the delivery of the implementation intervention in each service. The CMEs will train the staff, monitor performance, and conduct academic detailing with antenatal clinicians and service managers.
5. *Clinician training and educational resources* [30, 60]: Multi-mode (online and face-to-face) training will be provided to clinicians in each maternity service, facilitated by the CMEs. The staff will participate in 1–2 h of training during the intervention period. Face-to-face training sessions will be rostered into routine educational sessions. The training will focus on addressing local barriers to care delivery and use evidence-based training elements including role-plays and case studies [30]. Printed educational resources providing instructions on the gestational weight gain care pathway will also be provided.
6. *Care delivery monitoring and feedback (including academic detailing)* [31, 32]: Data from patient surveys and electronic medical records will be used to compile monthly reports on compliance with the delivery of the gestational weight gain care pathway. Service managers will be supported to set care delivery goals, monitor progress, and develop action plans in response to feedback. Performance measures will be built into existing monitoring and accountability frameworks.

##### Method to prioritise implementation strategies and tailor content of intervention strategies to each sector’s context

The following staged method will be undertaken at each of the three intervention sectors to prioritise implementation strategies and tailor the content of strategies to each sector’s context:

1. Quantitative anonymous surveys will be undertaken with antenatal care providers to determine priority barriers to their implementation of the gestational weight gain care pathway. The surveys will use a best-worst scaling method [61, 62] to elicit the priority barriers clinicians face in undertaking elements of the gestational weight gain care pathway. The barriers included as options for care providers to choose from will be selected based on prior formative work undertaken by the research team and existing literature [24].
2. The priority barriers identified through the surveys will be defined in terms of the Theoretical Domains Framework (TDF) [24, 36] and the Capacity, Opportunity, Motivation- Behaviours (COM-B) model [33] and then mapped using the Behaviour Change Wheel [33] to intervention functions and behaviour change techniques [25, 26].
3. Consultation with Aboriginal community members, Aboriginal Community Controlled Health Services within the participating sectors, AMIHS staff, and Aboriginal staff within the study team will be undertaken to ensure the content of implementation strategies is culturally appropriate.
4. Final refinement of implementation strategies and development of strategy content will be undertaken following consultation with key antenatal care providers and managers from each of the three participating sectors. All modifications made to the gestational weight gain care pathway and implementation intervention due to sector context and needs will be reported according to the FRAME-IS [63].

#### Implementation intervention delivery timeline

The implementation intervention will be implemented in each sector sequentially for 4 months prior to the follow-up data collection (Fig. 1). Given their organisational and system focus, all strategies, other than the dedicated Clinical Midwife Educator (CME), have the potential to continue to be implemented following the 4-month study intervention period, subject to the operational decisions of the local health district.

### Control and contamination

#### Usual care

Prior to implementation of the implementation intervention in each of the three sectors, usual antenatal care for gestational weight gain during pregnancy will be provided. Such care is likely to vary by maternity service and clinician.

#### Potential for contamination

As the research team will control the initiation and delivery of all the intervention elements, the intervention strategies will not be accessible to antenatal clinicians during the baseline (control) phase. Although the potential for contamination during this phase from staff movement between sectors is possible, it is likely to be limited due to the structural and organisational nature of the implementation strategies. Information on the movement of clinicians between participating sectors will be collected throughout the study.

### Measures

#### Primary trial outcome

The primary trial outcome is the proportion of all antenatal clinic appointments (at ‘first appointment’, 27–28 weeks gestation and, 35–36 weeks gestation) for which women report receiving the following:

1. An assessment of gestational weight gain using objective measures of weight against recommended weight gain targets
2. Advice on gestational weight gain, dietary intake, and physical activity
3. Offer of a referral to the NSW GHiP Service and, for women who are gaining weight above or below their recommended weight gain target*,* and where available, offer of a referral to a dietetics service (including culturally appropriate dietetics services for Aboriginal women)

#### Secondary trial outcomes

The following are the secondary trial outcomes:

1. The proportion of all antenatal clinic appointments (at ‘first appointment’, 27–28 weeks gestation, and 35–36 weeks gestation) for which clients report receiving all elements of the gestational weight gain care pathway (‘complete gestational weight gain care’):
  - An assessment of gestational weight gain using objective measures of weight against recommended weight gain targets
  - Advice on gestational weight gain, dietary intake, and physical activity
  - Offer of a referral to the NSW GHiP Service and, for women who are gaining weight above or below their recommended weight gain target, and where available, offer of a referral to a dietetics service (including culturally appropriate dietetics services for Aboriginal women)
2. The proportion of women attending antenatal clinic appointments (at ‘first appointment’, 27–28 weeks gestation, and 35–36 weeks gestation) that report accepting a referral to the NSW GHiP Service and, for women who are gaining weight above or below their recommended weight gain target, and where available, accepting a referral to a dietetics service (including culturally appropriate services for Aboriginal women)
3. The proportion of women that report weight gain within the recommended ranges at 28 weeks and 36 weeks gestation based on the IOM recommendations according to the four categories of pre-pregnancy BMI (underweight, healthy weight, overweight, and obese)
4. Estimated average rate of weekly weight gain at 28 weeks and 36 weeks gestation, according to the four categories of pre-pregnancy BMI (underweight, healthy weight, overweight, and obese)
5. Mean maternal diet quality score at 28 weeks and 36 weeks gestation calculated by an 11-item food frequency questionnaire based on the Australian Guide to Healthy Eating [52]
6. Mean total maternal physical activity levels (minutes/week) at 28 weeks and 36 weeks gestation assessed using the 7-item International Physical Activity Questionnaire (IPAQ) short form, which has been found to be both reliable and valid in assessing physical activity in adults [64]

#### Process measures

The acceptability, adoption, fidelity, and penetration/reach of the best practice care pathway for addressing gestational weight gain and the implementation strategies from the perspective of clients and clinicians will be measured. These process measures will be based on an implementation evaluation framework specified by Proctor et al. [65] and use validated measures where available [66]. Measures to assess the penetration/reach of the implementation intervention will include the participation of antenatal clinical staff in educational meetings, interaction of clinical staff with local opinion leaders, involvement in academic detailing/audit and feedback sessions, and receipt of clinical practice guidelines. To determine penetration/reach by different groups of clinicians, data will be collected from clinicians on position/profession, level of training, and length of time working in antenatal care. Contextual factors, including measures of the social, political, or economic environment that might influence implementation (e.g. changes due to COVID-19 restrictions), will also be recorded [67].

To assess the delivery of the care pathways to different demographic groups of women, the following information will be collected: age, gender, highest level of education, employment status, geographical location, Aboriginal or Torres Strait Islander status of woman and baby, household composition, current gestation, and gestation at the first antenatal appointment, whether attending care for their first or subsequent pregnancy and pre-pregnancy BMI.

#### Within-trial economic analyses

The within-trial analysis will investigate the cost-effectiveness of the implementation intervention through two different analyses. The primary analysis will be a cost-effectiveness analysis which will estimate the incremental cost per unit change in the primary trial outcome. Additionally, in complex public health evaluation research, it is questionable whether all the relevant impacts can be captured in a single economic summary measure. Hence, the use of cost-consequence analysis) is also recommended [68]. Secondary outcomes as well as disaggregation of the outcomes by sector will be included in the cost-consequence analysis. Both analyses will be conducted and reported in accordance with the Consolidated Health Economic Evaluation Reporting Standards (CHEERS) publication guidelines and good reporting practices guidelines [69]. A budget impact assessment will be conducted to inform the affordability of the implementation intervention from the perspective of the health service. In addition to measuring the efficiency of the implementation intervention, an analysis of equity impact will be conducted to assess whether any observed impacts/gains are equitably shared among the target population.

### Data collection procedures

#### Data for primary and secondary outcome measures

Each week, a sample of eligible women who have attended an antenatal clinic appointment in the last week will be selected and sent a letter providing information about the study and inviting them to participate in a computer-assisted telephone interview (CATI). Telephone contact will be attempted with women up to 10 times over a 2-week period, including at different times of the day and on weekdays and weekends, to elicit consent and complete the survey. If a woman declines to participate in the CATI, they will be invited to complete the survey online. If they consent to participate in the online survey, they will be sent a survey link via text message. Women who are of Aboriginal or Torres Strait Islander origin and/or are attending or enrolled to attend an AMIHS will be offered via text message the choice of participating in the survey via either CATI or online mode.

The CATI survey will be conducted by experienced female interviewers who will receive specific training, including practice interviews before they commence. The CATI and online survey script are identical in the wording of questions, response options, and help provided. Both surveys will be pilot tested prior to starting the study to test comprehension, logic flow, and survey length. These data collection procedures have been successfully used in past studies undertaken by the research team [70].

#### Data for process measures

Data for process measures will be collected via surveys with women (described above) and antenatal care providers. Online surveys of antenatal care providers will be conducted at the completion of the intervention in each sector. All eligible antenatal care providers at the participating sectors will be sent a link to an online survey via email as well as given the option to complete the survey on tablet computers in regular in-services and clinic meetings. Surveys will be completed anonymously. Additional process data be collected using project management logs completed by project staff.

#### Data for economic analyses

Data regarding resources expended on materials, labour, and other expenses incurred in developing and executing the implementation intervention will be recorded prospectively in project management logs. Such cost will include actual labour costs for training, managerial oversight, and all activities undertaken by the CMEs using salary awards. Costs will be reported in Australian dollars. Research and data collection and analysis costs will be excluded.

#### Overall data management

Management of trial data will be in accordance with a data management protocol that has been developed and approved by the project’s advisory group. Data will be stored securely as per the requirements of the Hunter New England Human Research Ethics Committee, the University of Newcastle Human Research Ethics Committee, and the Aboriginal Health and Medical Research Council. Data will only be accessible to the research team and statisticians. Confidential participant data will be stored securely and not linked to survey responses.

### Sample size and power calculations

Based on recruitment outcomes of previous trials conducted by the research team in clinical settings and maternity services specifically, it is expected that 70% of invited women, or 15 women (average of 5 per service), will consent to participate in weekly surveys for each of the three time periods (‘first appointment’, ‘27–28 weeks gestation’, or ‘35–36 weeks gestation) (total *N* = 45 per week) [70]. This will yield 5600 data points over the course of the data collection period, sufficient to detect (with 80% power and with a Bonferroni adjusted alpha of 0.0167) for the primary outcomes a (1) 15% absolute increase in receipt of recommended assessment of gestational weight gain (baseline prevalence estimate of 40%), (2) 14% absolute increase in recommended advice (baseline prevalence estimate of 30%), and (3) 9% absolute increase in the offer of recommended referral (baseline prevalence estimate of 10%).

### Statistical analysis

Pre- and post-intervention primary outcome data will be analysed using mixed effects logistic regression models to detect a change in the reported receipt of each of the three elements of the best practice care pathway for addressing gestational weight gain (assessment, advice, and referral) for all three sectors combined. Separate models will be fitted for each outcome. The main predictor of interest will be a before/after intervention indicator variable as well as a fixed effect for time, and a random intercept for an antenatal time point (first appointment, ‘27–28 weeks gestation’, or ‘35–36 weeks gestation’). The implementation intervention will be declared effective if the coefficient for the intervention period variable is below the threshold of *α* = 0.0167 for any of the three primary outcomes. A time-series analysis will be performed over the post-intervention periods to determine if slope changes occur, as an indicator of sustainability. Pre- and post-intervention secondary outcome data will be analysed using mixed effects logistic regression models to detect a change in each outcome for all three sectors combined. Descriptive statistics will be used to report on the process measures. The cost analysis and practice change cost-effectiveness results will be incorporated into an economic model to project the expected costs and outcomes that would be associated with broader scale-up of the implementation intervention to antenatal services across the state. SAS (V9.3 or later) will be used for all statistical analyses.

### Research trial governance

A research co-production approach has been employed in the development and design of the study [70]. The conduct of the study will similarly be overseen by an advisory group consisting of researchers, policymakers, practitioners, and clinical experts with expertise related to gestational weight gain, nutrition, physical activity, health promotion, implementation science, health economics, study design and statistics, Aboriginal health, obstetrics, and midwifery. A project team consisting of research staff and practitioners will develop and operationalise implementation strategies and data collection components of the trial according to the study protocol. Implementation leadership groups established within each of the three participating sectors will provide advice on the aspects of the gestational weight gain care pathway and implementation strategies that require sector-specific tailoring and oversee the delivery of the implementation intervention in their sectors.

Aboriginal people will be included at all stages of project governance, including the abovementioned advisory group, project team, and sector-specific implementation leadership groups. A series of Aboriginal cultural governance task groups, co-led by Aboriginal and non-Aboriginal staff, will provide guidance on cultural considerations for Aboriginal and Torres Strait Islander people relating to the gestational weight gain care pathway, implementation strategies, data collection, and interpretation and dissemination of study findings. A cultural review group containing only Aboriginal members will review all project resources and dissemination products.

### Trial discontinuation or modification

There are no predetermined criteria for trial discontinuation as it is not anticipated that any events would occur that would warrant discontinuing the trial. Any unforeseen adverse events will be reported to the Hunter New England Human Research Ethics Committee (primary approval committee) and advice sought regarding the required action. Any negative effects reported by women regarding the acceptability of weighing and the care they received for gestational weight gain will be monitored and actioned as appropriate. The trial registration record will be updated with any protocol modifications, and any deviations from the original protocol will be reported in study outcome papers.

## Discussion

The research outlined in this protocol will fill an evidence gap regarding the effectiveness of implementation strategies to improve antenatal care addressing gestational weight gain. The findings will directly address Australian policy and practice priorities and needs at a national [8], state [49, 71], and local level [72] and have the potential to be applied at scale in other jurisdictions internationally.

The stepped-wedge design is appropriate for conducting a trial across multiple maternity services where the gestational weight gain care pathway is being introduced as part of routine care at a service level. Study strengths include the theoretical framework and formative surveys used to develop the intervention content, the blinding of data collection staff and survey participants, and the cultural governance model applied to the project. A research co-production approach has been employed in the design of the study and will be employed in its conduct and dissemination, and all study processes and outputs will be reviewed for cultural appropriateness.

## Trial status

Protocol version 1. 4 August 2021. Recruitment of Sector (Site) 1 to commence on 1 September 2021. Recruitment of the last sector (site) to be completed in April 2022.

## Data Availability

Not applicable.

## Abbreviations

AMIHS: Aboriginal Maternal Infant Health Service
BMI: Body mass index
CATI: Computer-assisted telephone interview
CME: Clinical Midwife Educator
HNELHD: Hunter New England Local Health District
NSW: New South Wales
GHiP: Get Healthy in Pregnancy
